# Medical Opinion and Movement

**Published:** 1909-04-03

**Authors:** 


### Medical Opinion and Movement.

A CASE of Diabetic Gangrene affecting the breast is reported in the *Australasian Medical Gazette* by Dr. Swift, of Adelaide. The onset of the condition was very sudden, with agonising pain, and in the middle of the night. The pain was referred to the left nipple and was described as similar to that of a red-hot iron being bored into the skin. One and a half inches below and to the outer side of the nipple was a livid bluish spot, which in a few hours was gangrenous and covered with blebs. The temperature was 102.6° F. and the pulse rate 120. There was a very large quantity of sugar in the urine, and diacetic acid also; but no albumin was present. For some days the patient's condition was serious: vomiting was very troublesome, pain intense and subdued only by morphia, and fever up to 104°. Subsequently a new focus of gangrene appeared near the sternum, and the slough demarcated around the original patch measured six inches by four. Ultimately the two foci ran into one, which healed up satisfactorily, and simultaneously the sugar disappeared from the urine, which became normal also in quantity and in specific gravity. It is known that a severe fever, as, for example, enteric, affecting a diabetic patient will sometimes be accompanied by disappearance of sugar from the urine, and Dr. Swift suggests that the similar result in his case may be ascribed to the products of the growth of the micro-organisms in the sloughing masses of breast-tissue. He has searched the literature for a similar case, but is unable to find any record of one: certainly diabetic gangrene affecting an organ so near the heart and so well provided with blood-vessels must be extremely rare.

THE Treatment of Erysipelas by carbolic acid and alcohol is warmly advocated by Dr. Judd in the *Medical Record*. The entire affected surface is swabbed with a 95 per cent. solution of carbolic acid, and also some half an inch of the surrounding healthy skin. This is left on until the reddened colour of the inflamed area is replaced by the whitening due to the acid, and the latter is then swabbed off with pure alcohol until a pink colour is regained. If a large portion of skin is involved, it is advisable to do one section of it at a time; but the whole may be treated by successive applications at one sitting. The result of this system is immediate and complete cessation of the burning and itching sensations which distress patients so much. Within twenty-four hours the temperature sinks to normal, appetite returns, and swelling, if not quite lost, is at least much diminished. The whitening caused by the carbolic acid must not be allowed to go on to thorough blanching, otherwise a burn and subsequent slough will be produced. The extension to the neighbouring apparently sound skin is a highly important part of the treatment. It is said that no scarring results; the superficial layers of the skin merely come off as after mild sunburn, and the skin beneath is but slightly tender. Moist dressings soaked in saline solution or 1 in 2,000 perchloride of mercury solution are recommended as local after-treatment. Dr. Judd has tried the method in 82 cases, and reports 5 failures, 10 delayed recoveries, and 67 complete successes: he is convinced that it is very valuable in all kinds of cases, mild and severe.

AT the New York Obstetrical Society early this year, Dr. Ralph Waldo narrated a case which led to a lengthy discussion of a point that deserves attention at the hands of medico-legal experts. Dr. Waldo was called in to attend a woman, three feet of whose large intestine had been pulled out through a Rupture in the Uterus produced by a physician who had undertaken to empty the latter for inevitable abortion. The actual injury to the uterine wall was caused by an ovum forceps. The gut was immediately recognised and pushed back, and the uterus packed with iodoform gauze. On opening the abdomen a rent was found in the uterus two inches long, and bleeding freely; this was stitched up. The large intestine was completely torn across at the junction of the rectum with the sigmoid flexure, and the mucous membrane of the latter and of much of the descending colon had been pulled out and was found prolapsed in the peritoneal cavity. There were also several holes in the peritoneal coat of the colon. The damaged gut was resected, and the ends anastomosed with Murphy's button. Owing, it is believed, to the thorough preparation of the patient for the curetting, whereby the large intestine was practically empty, the subsequent course was entirely favourable, and the patient left hospital in three weeks.

THIS remarkable case was followed by the narration of similar accidents within the experience of the various gynæcologists who contributed to the debate. In fact, there appeared to be hardly anyone who had not at some time or other perforated the uterus, and many confessed also to having pulled intestine out of it. This series of confessions recalled to one speaker an action for *mala praxis* brought in this country in 1876 against a country practitioner, to whom the same catastrophe happened. In that case the doctor cut off six feet of intestine and threw it away; and a very eminent gynæcologist of the day who was called for the defence swore that such an event might happen in his own practice, with the result that the practitioner was acquitted. Now, in regard to another such action, should one ever arise, it may be very important to show that several of the foremost gynæcologists in New York have actually pulled down intestines, through a perforated uterus, though none of them went so far as to cut off the loop. One speaker had perforated the uterus four times whilst curetting, though he had never pulled abdominal contents through the hole. The other important fact which emerges is that several of those present felt that the dilators used were responsible for the uterine ruptures. Apparently a Sims dilator, and a modification of it known by the name of Wylie, are popular instruments for this purpose in America, and to the blades of these the damage is attributed. Possibly the use of Hégar's dilators explains the infrequency of these occurrences in Britain, if they really are infrequent, and not merely unreported.

OBSTETRIC opinion abroad as to the proper conduct of labour in various degrees of Pelvic Deformity seems to be by no means unanimous. Two papers by French obstetricians which have appeared lately emphasise these differences of practice. Pinard, in the *Bulletin Médicale*, sums up the teaching of the Baudelocque. He never induces labour, whatever the deformity, and also interdicts absolutely both forceps and version whenever the fœtus is alive. Cæsarean section, pubiotomy, and symphysiotomy are alone relied on for labour with contracted pelvis. The latter operation is never to be done until dilatation of the cervix is complete; and Cæsarean section is not undertaken until labour has set in. Labusquière, on the other hand, publishes in the *Annales de Gyn. et d'Obst.* the teachings of the Vienna school. Spontaneous delivery is waited for as long as possible. Version is done much more often than forceps operations, and prophylactic podalic version is a recognised method of treatment, either with or without the induction of premature labour. The high forceps operation is considered permissible in selected cases. Premature labour is induced, preferably at the end of the ninth lunar month, for conjugates from 7 to 8.5 cm., when previous labours have shown the impossibility of spontaneous delivery. Again, in Bumm's clinic at Berlin premature labour is now never induced. When labour begins, unless the pelvic contraction is extreme Bumm waits until it seems certain that help will be necessary. He then, under anæsthesia, makes a complete examination and proceeds to the necessary measures. These include version for slight contraction; pubiotomy for conjugates of 7 to 9 cm., when dilatation is complete; extra peritoneal Cæsarean section for conjugates of 6 to 6½ cm., and the classical operation for smaller ones still. He allows forceps for multiparæ after pubiotomy. Markedly as Continental obstetric practice differs from that taught in Britain, it is evident that divergences in different clinics are as great as, or greater than, even those between British and foreign views.

DR. DAWBARN, Professor of Surgery in the New York Hospital, draws attention to a valuable sign in cases of Fractures of the Long Bones, which he terms "osteophonic percussion." The sign is elicited in the following way: In the case of the humerus, for instance, a stethoscope is applied over the greater tuberosity, and, at the same time, an assistant percusses the external condyle. In an ordinary fracture with the ends in apposition little difference is noted in intensity and pitch of note from that produced by the sound humerus on the other side, but if fascia, muscle, or other soft tissue is interposed between the broken ends, the sound conduction is distinctly lessened and the pitch of the note lowered. As the author points out it is of the utmost importance that such a condition be determined at the earliest possible moment, so that it can be rectified, if need be, by operation, instead of first putting the limb up in splints, and discovering only after some weeks that the ends fail to unite owing to this interposition of soft tissue. He expresses surprise that this sign of osteophonic percussion has not found its way into any of the text-books on surgery, as he has found it of the greatest assistance in a large number of cases. He points out that a thin piece of interposed muscle or fascia will render hopeless bony union, and yet one may still be able to get crepitus perfectly well and consequently be entirely misled as to the condition.

"COLOUR-BLINDNESS.—Can it be remedied?" Such is the heading to an article by Dr. Brawley, of Salisbury, U.S., in the *International Journal of Surgery*. It is generally held that colour-blindness is due to an organic defect in the visual apparatus which is permanent and unalterable, and it is rather surprising to learn from this author that by diligent application and study the defect can apparently be completely overcome. He relates the case of a conductor on the Southern Railway whom on examination he found to be so colour-blind that he was unable to distinguish in the skein test green, blue, grey, brown, and different shades of red, and said they were all the same colour, differing only in shade. He was in consequence compelled to relinquish his post. He set to work, however, to study the colours and supplied himself with all the different colours of woollens and various samples of paint, and also provided himself with a miniature lantern. At the end of five weeks he was able to undergo the most rigid examination without a mistake, and later on he was also examined by the chief surgeon and two other oculists with the same result. Since then Dr. Brawley has encouraged several other colour-blind applicants to study to overcome their defect, and they have been able to do this in the same way. The author is, therefore, led to the conclusion that with practice the intelligent colour-blind can educate himself sufficiently to call ordinary colours correctly. Although he does this, it is probable that he does not see them as they really are or as others see them, but has, as it were, a standard of his own by which he makes the necessary distinctions.

DR. W. L. HARRIS, of Providence, U.S., advocates Mercurial Injections after the removal of Cancerous Growths as a means of treatment for secondary enlarged glands. With them he combines injections of a soluble iron salt and arsenic as tonics to counteract the tendency of the patient to anæmia and debility both from the disease and from the mercurial treatment. For the first two months after operation he injects every ten days 15 centigrams of salicylate of mercury, and then smaller doses every 20 days for the rest of the year. At the same time he injects .03 centigrams of a soluble iron salt and .0025 decimilligrams of arsenious acid at first every day and then at variable intervals. During the second year he continues these injections at longer intervals. This treatment appears to have given some excellent results in the hands of this author. In several cases, in which removal of affected glands was for one reason or another not undertaken at the time of operation, the glands have completely disappeared in the course of the treatment, and no recurrence of any kind has taken place. Such are cases of breast cancer with axillary and clavicular glands affected but not removed at the time of operation, and a case of cancer of the floor of the mouth with glands in the neck. The glands in this case were left for a subsequent operation, but disappeared rapidly under treatment with injections. The patients must of course be watched for signs of mercurial saturation, and blood estimations should be made to control any tendency to anæmia that may result from the mercurial injections.

---

Dr. Tedeschi has drawn attention to a peculiar Scapular Bruit, which he has observed many times in the course of examination of the chest. In order to elicit the phenomenon the patient is requested to breathe deeply or to move the arms to and fro. If, at the same time, the palm of the hand is placed upon the scapular region a peculiar sensation is perceived intermediate between a thrill and a rub. If the ear is applied to the same part a bruit is heard of the nature of a crepitation. This crepitation is heard most distinctly sometimes at the internal border of the scapula and sometimes in the supra-spinous fossa. The patient frequently calls the attention of the physician to the part on account of pain experienced, which is increased by palpation of the muscles. The author considers that the phenomenon is of muscular origin, and is due to an inflammatory process localised in the muscles of the scapular girdle and in the aponeuroses. He is of opinion that tuberculosis and excessive use of the muscles of the shoulders are both ætiological factors in the condition. In the course of the last three years he has found the condition in 58 patients, and of them 49 presented signs of tuberculosis. On the other hand, a large proportion of these patients were engaged in some employment such as dressmaking with the use of the hand sewing-machine, tailoring, washing, and bread-making. The so-called "professional" factor appears to be the more plausible and probably the more direct causal factor, and it is easy to understand that in subjects debilitated by tuberculosis excessive use of any group of muscles might occasion a lesion of the nature described. It does not seem necessary, however, to assume that the lesion is directly connected with the pulmonary trouble in the sense of its being of a tubercular nature. In fact, the author admits that some of the patients were suffering from anæmia and general debility.

---

Two cases of the rare condition known as Schlatter's Disease are reported by Dr. Bowser and Mr. Thomson respectively in the *Edinburgh Medical Journal*. Although not often reported it seems probable that the lesion is not so much rare as unfamiliar, and a knowledge of its existence and of the favourable prognosis associated with it would have saved the parents of Mr. Thomson's patient much unnecessary expense and anxiety. Schlatter's disease occurs chiefly in active boys about thirteen to fifteen years of age; it has also been described by Stimson under the name of avulsion of the tubercle of the tibia. The symptoms are pain in the region of the insertion of the patellar tendon, tenderness in the same spot, and sometimes lameness and discomfort after sitting for any length of time with the knee flexed in the usual way. Skiagrams show a solution of continuity in the bony downward prolongation of the epiphysis which normally forms, as will be remembered, from but one centre of ossification for both the tubercle and the epiphyseal end of the bone. The gap between the tongue-like process and the epiphysis itself is probably filled with cartilage; and it is believed that the lesion is caused by the powerful action of the quadriceps femoris acting through the patellar tendon on a tubercle in which a separate centre of ossification has formed. The want of continuity is not evidence of fracture, which is a totally distinct condition. Sir James Paget thought that the lesion represents "one of the least degrees of periostitis due to strain." No treatment is required.

---

A recent number of *La Presse Medicale* contains an account of a new reaction obtained by applying Tuberculin to the Nasal Mucous Membrane. The authors propose to use their method—which they claim to be quite free from danger—in place of Calmette's ophthalmo reaction. The technique of the method consists in placing a pledget of cotton-wool, soaked in a one-per-cent. Calmette solution, in contact with the mucous membrane of the lower part of the anterior nares for ten minutes. In from eighteen to forty-eight hours the mucous membrane becomes congested, and a local exudate can be seen, which rapidly dries up, leaving a thin yellowish crust behind. This crust falls spontaneously in about five days, the underlying mucous membrane being slightly congested. All the patients examined who were suffering from pulmonary tuberculosis gave a positive reaction, and out of seventy-three obviously tuberculous individuals sixty-eight positive reactions were obtained, and of the remaining five cases two showed congestion of the mucous membrane without exudation, the remaining three cases being lost sight of. The rhino-reaction would therefore seem to be as trustworthy as the ophthalmo- and cuti-reactions; it can, moreover, be used without the patient's knowledge, and in addition it has so far been free from any disagreeable consequence to the patient.

---

Lehmann, in the *Deutsche Milit. Zeit.*, describes a method of treating Ingrowing Toe-nails by local applications of perchloride of iron. It consists in painting the cutaneous overgrowth on its external and deep surfaces, as well as the ingrowing portion of the nail with a wooden stick, one end of which is wrapped round with cotton-wool soaked in a strong solution of perchloride of iron. These applications are repeated once daily, treatment as a rule being necessary for about a fortnight. The overgrowth quickly shrivels, hardens, and the nail softens. Pain is quickly abolished, and in mild cases the patient is able to get about as usual, providing he wears boots which are sufficiently roomy. In the more serious cases, when the part has become infected, a few days' rest are necessary.

